# Antimicrobial Resistance Profiles in *Enterococcus* spp. Isolates From Fecal Samples of Wild and Captive Black Capuchin Monkeys (S*apajus nigritus*) in South Brazil

**DOI:** 10.3389/fmicb.2018.02366

**Published:** 2018-10-09

**Authors:** Tiela Trapp Grassotti, Dejoara de Angelis Zvoboda, Letícia da Fontoura Xavier Costa, Alberto Jorge Gomes de Araújo, Rebeca Inhoque Pereira, Renata Oliveira Soares, Paulo Guilherme Carniel Wagner, Jeverson Frazzon, Ana Paula Guedes Frazzon

**Affiliations:** ^1^Microbiology, Immunology, and Parasitology Department, Institute of Basic Health Sciences, Federal University of Rio Grande do Sul, Porto Alegre, Brazil; ^2^Gram-Positive Cocci Laboratory, Federal University of Health Sciences of Porto Alegre, Porto Alegre, Brazil; ^3^Brazilian Institute of Environment and Renewable Natural Resources, IBAMA, Brasília, Brazil; ^4^Institute of Food Science and Technology, Federal University of Rio Grande do Sul, Porto Alegre, Brazil

**Keywords:** Enterococcus, primates, wild and captive capuchin monkeys, *Sapajus nigritus*, antimicrobial resistance

## Abstract

The environment, human, and animals play an important role in the spread of antibiotic-resistant bacteria. Enterococci are members of the gastrointestinal tracts of humans and animals and represent important reservoirs of antibiotic resistance genes. Until today, few studies have examined antibiotic susceptibility in enterococci isolated from primates. Therefore, the present study investigated species distribution, antibiotic susceptibility, and resistance genes in enterococci isolated from wild and captive black capuchins monkeys (*Sapajus nigritus*) in Rio Grande do Sul, South Brazil. A total of 24 swabs/fecal samples were collected, including 19 from wild monkeys living in two forest fragments [São Sebastião do Caí (SSC) and Santa Cruz do Sul (SCS)], and five in captive [Parque Zoológico da Fundação Zoobotânica (ZOO)], between August 2016 and November 2017. Fifteen colonies were randomly selected from each sample. Enterococci were identified by MALDI-TOF, tested for susceptibility to 12 antibiotics; and screened for *tet*(S), *tet*(M), *tet*(L), *msrC*, and *erm*(B) genes by PCR. Two-hundred ninety-six enterococci were isolated (SSC *n* = 137; SCS *n* = 86; ZOO *n* = 73) and differences in *Enterococcus* species distribution were detected on three monkey groups, with low abundance in SCS (1 - D = 0.2), followed by ZOO (1 - D = 0.68), and SSC (1 - D = 0.73). The enterococci frequently recovered include the following: *Enterococcus faecalis* (42.6%), *E. hirae* (29.1%), and *E. faecium* (15.9%). Antibiotic-nonsusceptible was observed in 202 (67.9%) strains. The rate of non-susceptibility to rifampicin, tetracycline, erythromycin, nitrofurantoin, chloramphenicol, and ampicillin was 46%, 26%, 22% and 19%, 13%, 0.3%, and 0.3%, respectively. All strains were susceptible to vancomycin, streptomycin, gentamycin, and linezolid. Forty-three (14.52%) isolates were identified as multidrug resistant (MDR), and the highest number of MDR enterococci were *E. faecium* recovered from wild monkeys living close to a hospital and water treatment plant. Elevated rates of antibiotic resistance genes *msr*C and *tet*(L) were isolates from ZOO. In conclusion, differences in the frequency of enterococci species, antibiotic-nonsusceptible and antibiotic resistance genes in all groups of monkeys were identified. These data suggest that anthropogenic activities could have an impact in the resistome of primate gut enterococci communities.

## Introduction

Brazil has the greatest biodiversity on the planet, comprising approximately 103,870 different animal species and the highest diversity of Primates, around 77 species, including the howler monkey, the capuchin monkey, the marmoset, and the tamarin (Brazilian Society of Primatology [SBP], 2016). *Sapajus nigritus* (black-horned capuchin or black capuchin monkeys) are part of the Cebidae family, characterized as robust capuchin monkeys with adornments or tufts on the head (Rylands et al., 2012). They are considered the largest omnivorous Neotropical primate, which is able to adapt its diet according to food availability, thus bringing them into contact with a wide diversity of microorganisms. Their diet is composed of approximately 55% fruits, 33% insects, 8% seeds, 8% leaves (mainly young), and 2% flowers (National Research Council of the National Academies [NRC], 2003). Currently, this species occurs in Minas Gerais, Rio de Janeiro, São Paulo, Paraná, Santa Catarina, and Rio Grande do Sul states, extending to the Argentinean province of Misiones (International Union for Conservation of Nature [IUCN], 2017).

The black capuchin monkeys (*S. nigritus*) live in different habitats, from large remnants or continuous to small forests fragments. Outside of their natural environment, they can be found in zoological, rehabilitation, or research centers, and even in urban and rural environments. Additionally, these animals exhibit a niche overlap with humans in the case of semi-wild areas (Muehlenbein, 2017). Since the natural habitats of primates are forests, most interactions between humans and primates occur in this high-risk interface. In many regions of the world, omnivorous primate species are adapting to human activities. Furthermore, the frequency of such interactions has increased due to ecotourism and/or increasing forest invasion, and these interactions could lead bacteria exchanges by multiple routes, namely through the offering of food (Mikich and Liebsch, 2014). Glover (2014) compared the enteric bacteria of monkeys with three levels of human contact and determined that the closer the animals were to humans, the more resistant was the enteric bacteria to antibiotics. Importantly, Rolland et al. (1985) observed that wild baboons (*Papio cynocephalus*) that fed on human debris, maintained a high proportion of antibiotic-resistant enteric bacteria than those without human contact.

The environment, humans, and animals play an important role in the emergence and spread of antibiotic-resistant bacteria. Singer et al. (2016) described three well-characterized classes of chemicals – antimicrobials, heavy metals, and biocides – related to the selection of antibiotic resistance genes. Biological fluids (e.g., urine and feces) contaminated with antimicrobials or resistant bacteria from human and animal origins are released into the environment – especially in soil, sewage, water, and wastewater – thereby contributing to the spread of resistance (Baquero et al., 2008; Gothwal and Shashidhar, 2014). The proximity to human activity has showed to increase the number of resistant bacteria in wild animals, with animals living near waste or agricultural water harboring more antibiotic-resistant bacteria than animals living close by unpolluted water (Allen et al., 2010). Recently, it was demonstrated that exposure to human antibiotics was associated with changes in the microbiota composition of baboons (Tsukayama et al., 2018).

Enterococci are a large genus of bacteria widely distributed on plants, soil, water, humans, and animals. In humans and other species, inhabit various sites including the oral cavity, genitourinary and gastrointestinal tracts (Lebreton et al., 2014). The genus *Enterococcus* consists of over 50 diverse species, and *Enterococcus faecalis*, *E. faecium*, *E. hirae*, *E. durans*, *E. casseliflavus*, *E. gallinarum*, and *E. mundtii* are the most frequently encountered in the gastrointestinal tracts of animals (Poeta et al., 2005; Cassenego et al., 2011; Lozano et al., 2016; Medeiros et al., 2017). However, the species evaluation in the gastrointestinal tract of primates remains little known (Xavier et al., 2010; Glover, 2014). The species distribution, as well as their proportions in the different niche can change according to the host and its age, diet, underlying diseases, and prior antimicrobial therapy (Lebreton et al., 2014).

Otherwise, enterococci are considered an opportunistic pathogen, associated with serious infection, such as endocarditis, urinary, and bloodstream infections, intra-abdominal end intra-pelvic abscesses, which has been attributed, in part; to the increasing resistance to a wide range of antimicrobial agents. The presence of resistant and multidrug-resistant enterococci in patients has a clinical relevance because of limited therapeutic options (Higuita and Huycke, 2014). Antimicrobial resistance to several classes of agents is a remarkable characteristic of enterococcal isolates. These microorganisms are intrinsically resistant to some antimicrobial agents commonly prescribed for Gram-positive cocci, and exhibit resistance to a wide variety of other antimicrobials by mutation and/or acquisition of genes through the plasmids and transposons. In fact, many species are recognized for their ability to acquire and transfer resistance and virulence genes, which give a selective advantage to *Enterococcus* spp. survival and dispersion in the environment (Lebreton et al., 2014; Miller et al., 2014). The occurrence of antimicrobial resistance among enterococci is not restricted to the nosocomial setting, and therefore, resistant strains has been investigated and monitored in different habitats, providing important information regarding about environmental disturbances (Poeta et al., 2005; Frazzon et al., 2010; Barros et al., 2011; Cassenego et al., 2011; Santos et al., 2013; Santestevan et al., 2015; Prichula et al., 2016).

To date, few studies have examined the presence of enterococci in monkeys, and these studies have focused primarily on captive animals, perhaps due to the inherent difficulty in obtaining samples from free-living wild animals (Xavier et al., 2010; Glover, 2014; Woods et al., 2017). The investigation of the persistence of enterococci in these animals highlights the impact of human activities on the environment. Moreover, antibiotic-resistant enterococci in monkeys are an important point that must be addressed in the host–microorganism–environment interactions. Therefore, the objective of the present study was to evaluate the distribution of enterococci in fecal samples of free-living and captive black capuchin monkeys from South Brazil. In addition, the prevalence of antibiotic susceptibility and antibiotic resistance genes in enterococci isolated from these primate populations were determinates.

## Materials and Methods

### Sample Collection

Twenty-four samples collected from black capuchin monkeys between August 2016 and November 2017 were used in the present study, including samples from animals with free lifestyle (*n* = 19) and animals living in captivity (*n* = 5). Samples were obtained in Rio Grande do Sul, South Brazil (**Supplementary Data 1**).

**FIGURE 1:**
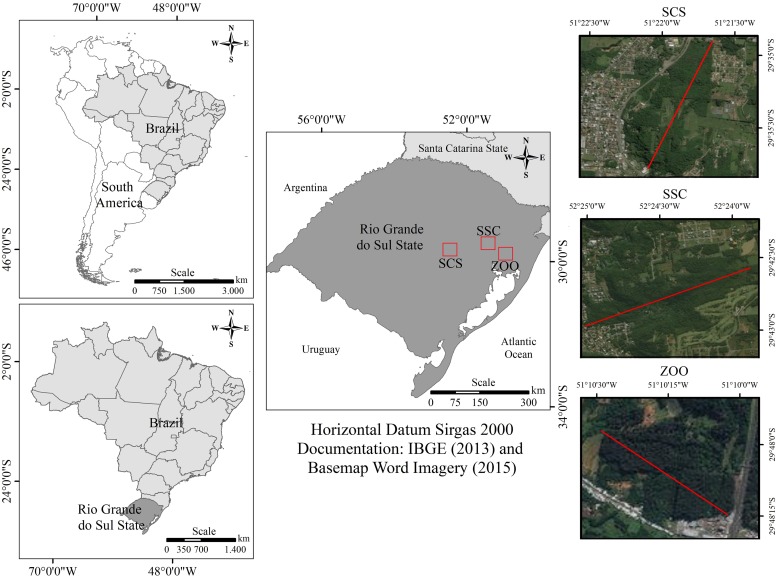
On the left Southern American (top) and Brazil (bottom) maps. On the center, Rio Grande do Sul map showing locations of collected samples site. On the right-detailed map of the Rio Grande do Sul showing collected samples locations: Top; SSC, São Sebastião do Caí – a fragmented forest coverage of 2% vegetation of Atlantic Forest, totaling 9611 hectares of forest: Center; SCS, Santa Cruz do Sul – a fragmented forest comprised of 13% vegetation, totaling 539.8 hectares of forest; Botton; ZOO, Zoological (ZOO) at Zoobotanical Foundation of Rio Grande do Sul, in Sapucaia do Sul city.

Samples were taken from three groups of black capuchin monkeys. Two groups include wild animals from two forest fragments in Rio Grande do Sul State (**Figure 1**). In the first forest fragment located in São Sebastião do Caí (SSC) (29° 35′ 13″ S; 51° 22′ 17″ W), samples were obtained from 11 animals, corresponding to 30% of overall group composition. This forest fragment is located near to a hospital and water treatment plant. The area comprises 2% of vegetation, totalizing 9611 hectares of forest (SOS Mata Atlântica, 2016). In the second forest fragment, located in *Parque Municipal da Gruta dos Índios* (Indian Grotto Municipal Park) in Santa Cruz do Sul (SCS) (29° 43′ 03″ S; 52° 25′ 33″ W), samples were obtained from eight animals, corresponding to 27% of the overall group. This forest fragment is located inside of the park, and the animals come without indirect contact with any park visitor, but maintain contact with garbage and other food sources. The area comprises 13% of vegetation, totalizing 539.8 hectares of forest (SOS Mata Atlântica, 2016). The third group was in captive condition at the Zoological Park of the Zoobotânica Foundation of Rio Grande do Sul (ZOO) in Sapucaia do Sul (29° 49′ 29″ S; 51° 08′ 54″ W), and five samples were collected. The animals were isolated in quarantine at ZOO since they were rescued from illegal or abusive situations by the Wild Animals Triage Center (CETAS – IBAMA). The diet of captive monkeys was composed of extruded ration for primates (Nuvital Primatas Neotropicais, Nuvital Nutrientes S/A, Colombo, Brazil) complemented with fruits and vegetables.

Wild capuchin monkeys were captured and manipulated using conventional methods according to the protocol for sample collection described by Instituto Chico Mendes de Conservação da Biodiversidade [ICMBio] (2012) using Tomahawk-type cages. The ketamine (100 mg/mL) and xylazine (20 mg/mL) were used intramuscularly for wildlife immobilization (Miranda et al., 2011).

Rectal swabs and fecal samples were collected by veterinarians, all animals were clinically healthy and were classified according to gender and age group. Rectal swabs were collected from the perirectal area, stored in Stuart transport medium (Kasvi, Paraná, Brazil), and transported to our laboratory for microbiological analyses. Fecal samples were collected, individually or in groups, directly from cages using sterilized wooden sticks. Fecal samples were placed in sterile tubes, kept on ice, and sent to our laboratory for storage at -80°C.

This study was carried out in accordance with the recommendations of Brazilian Institute of Environment and Renewable Natural Resources (IBAMA) and Chico Mendes Institute for Biodiversity Conservation (ICMBio). The protocol was approved by Information Authorization System in Biodiversity (SISBIO) number 56640.

### Isolation and Identification of Enterococci

Isolation, enumeration, and characterization of enterococci in fecal/rectal swabs were performed as previously described by Prichula et al. (2016) and Santestevan et al. (2015). Swabs or fecal samples (0.1 g) were inoculated in 9 mL of azide dextrose broth (Himedia, Mumbai, India) and incubated for 24 h at 37°C. Aliquots of 1 mL were placed in 9 mL of saline water, and initial samples were further diluted 10-fold to obtain a final dilution factor of 1/1000. From each dilution, 100 μL was inoculated in brain heart infusion (BHI) agar plates (Himedia, Mumbai, India) supplemented with 6.5% NaCl, before being incubated as previously described (Santestevan et al., 2015; Prichula et al., 2016). Fifteen colonies were randomly selected from each sample. Phenotypic criteria, such as size/volume, shape, color, gram staining, catalase production, growth capacity at 45°C, and bile aesculin reaction were used to separate the enterococci group and the non-enterococcal strains. Selected pure colonies were stored at -20°C in a 10% (w/v) solution of skim milk (Difco, Sparks, MD, United States) and 10% (v/v) glycerol (Neon Comercial Ltda, São Paulo, SP, BR).

The isolates collected were identified using matrix-assisted laser desorption and ionization time-of-flight technique (MALDI-TOF) applied to *Enterococcus* spp. according to the protocol previously described by Sauget et al. (2017).

### Antimicrobial Susceptibility Testing

Susceptibility to antimicrobial agents was performed using the Kirby–Bauer disk diffusion method recommended by the Clinical and Laboratory Standards Institute (Clinical and Laboratory Standards Institute [CLSI], 2016). Twelve antibiotics commonly used in clinical and veterinary medicine were evaluated: ampicillin 10 μg (AMP), ciprofloxacin 5 μg (CIP), chloramphenicol 30 μg (CHL), erythromycin 15 μg (ERY), streptomycin 300 μg (STR), gentamicin 120 μg (GEN), linezolid 30 μg (LNZ), nitrofurantoin 300 μg (NIT), norfloxacin 10 μg (NOR), rifampicin 5 μg (RIF), tetracycline 30 μg (TET), and vancomycin 30 μg (VAN). Minimum inhibitory concentration (MIC) of linezolid was determined by broth microdilution and interpretation of the results was performed following CLSI guidelines.

*E. faecalis* ATCC 51299 and *E. faecium* ATCC 53519 were included as control strains.

Strains resistant to three or more unrelated antibiotics were considered as multidrug-resistant (MDR). Intermediate and resistant strains were considered in a single category and classified as antibiotic-nonsusceptible.

### Detection of Resistance-Related Genes in *Enterococcus* sp.

DNA extraction was performed as described by Depardieu et al. (2004). PCR was carried out for the detection of six different resistance-related genes commonly observed in clinical and environmental enterococci, namely, *erm*(B), *msr*C, *tet*(M), *tet*(S), and *tet*(L) (Sutcliffe et al., 1996; Aarestrup et al., 2000; Werner et al., 2001; Frazzon et al., 2010; Rathnayake et al., 2011). *erm*(B) encodes a ribosomal methylase that mediates macrolides, lincosamides, and type B streptogramins resistance; *msr*C encodes for a macrolide and streptogramin B efflux pump; *tet*(M) and *tet*(S) encodes for tetracycline resistance via a ribosomal protection protein mechanism; and *tet*(L) encodes for tetracycline resistance via efflux pumps proteins.

### Statistical Analysis

The correlation between antimicrobial susceptibility presented by *Enterococcus* spp. and monkey collection origins were analyzed using a cross-table with Pearson’s chi-square test (χ^2^) (*p* ≤ 0.05) and Fisher’s exact test for small samples (≤5). Simpson’s index of diversity (D) was calculated to assess the differentiation of enterococci species among the monkeys from the different locations (Hunter and Gaston, 1988).

## Results

### *Enterococcus* spp. Isolation and Identification in Fecal Samples

The distribution of *Enterococcus* species recovered from fecal/rectal samples of wild and captive black capuchins monkeys is provided in **Table 1**. A total of 296 enterococci were isolated, of those 223 (75%) were recovered from wild (SSC *n* = 137; SCS *n* = 86), and 73 (25%) from captive monkeys (ZOO). Among enterococci isolated, *E. faecalis* (42.6%; *n* = 126), *E. hirae* (29.1%; *n* = 86), and *E. faecium* (15.9%; *n* = 47) were detected in all groups of monkeys; and *E. durans* (6.8%; *n* = 20), *E. casseliflavus* (4.4%; *n* = 13), *E. raffinosus* (0.3%; *n* = 1), *E. avium* (0.3%; *n* = 1), *E. gallinarum* (0.3%; *n* = 1), and *Enterococcus* sp. (0.3%; *n* = 1) were occasionally detected in the animals.

**Table 1:** Species distribution of enterococci in fecal samples of wild and captive black capuchin monkeys (*Sapajus nigritus*).

Species	Number (%) of Enterococci Isolated From			
	SSC	SCS	ZOO	Total (%)
*E. faecalis*	44 (32.1)	77 (89.5)	5 (6.8)	126 (42.6)
*E. hirae*	49 (35.8)	2 (2.3)	35 (47.9)	86 (29.1)
*E. faecium*	26 (19.0)	2 (2.3)	19 (26.0)	47 (15.9)
*E. durans*	7 (5.1)	–	13 (17.8)	20 (6.8)
*E. casseliflavus*	9 (6.6)	4 (4.7)	–	13 (4.4)
*E. raffinosus*	1 (0.7)	–	–	1 (0.3)
*E. avium*	1 (0.7)	–	–	1 (0.3)
*E. gallinarum*	–	–	1 (1.4)	1 (0.3)
*Enterococcus* sp.	–	1 (1.2)	–	1 (0.3)
Total	137 (100)	86 (100)	73 (100)	296 (100)


*SSC, São Sebastião do Caí; SCS, Santa Cruz do Sul; ZOO, Sapucaia do Sul.*

Differences in the distribution of *Enterococcus* spp. was detected amongst the three groups of black capuchin monkeys, as shown in **Table 1**. Samples from SSC presented the higher difference and relative abundance of enterococci, when compared to SCS and ZOO. The Simpsons diversity indexes showed differences between the three groups, with low abundance to SCS (1 - D = 0.2), followed by ZOO (1 - D = 0.68) and SSC (1 - D = 0.73). *E. faecalis* was the predominant species recovered from wild monkeys from SCS (89.5%; *n* = 77). On the other hand, in fecal samples of wild monkeys from SSC, the more commonly observed species were *E. faecalis* (32.1%; *n* = 44), *E. hirae* (35.8%; *n* = 49), and *E. faecium* (19.0%; *n* = 26). Whereas *E. hirae* (47.9%; *n* = 35), *E. faecium* (26.0%; *n* = 19), and *E. durans* (17.8%; *n* = 13) were the most abundant species isolated in fecal samples of captive monkeys.

### Antimicrobial Susceptibility Profile

**FIGURE 2:**
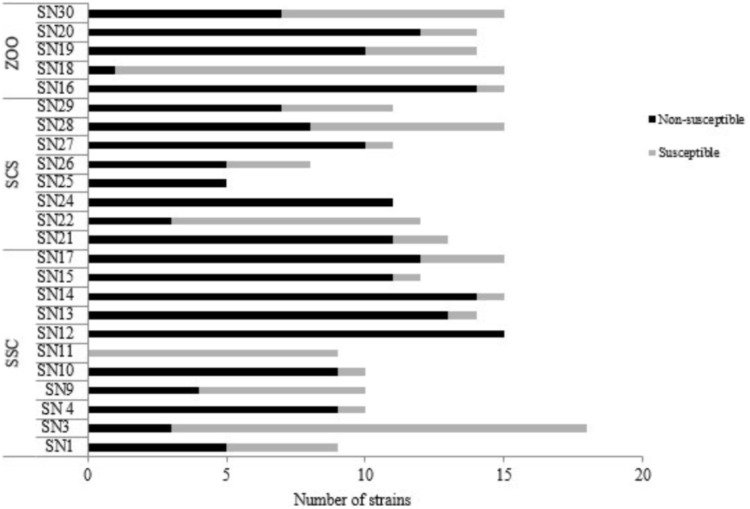
Numbers of susceptible and nonsusceptible enterococci species isolated from fecal samples of wild (SSC and SCS) and captive (ZOO) black capuchin monkeys (*Sapajus nigritus).* SN, identification sample of black capuchin monkeys. SSC, São Sebastião do Caí; SCS, Santa Cruz do Sul; ZOO, Sapucaia do Sul.

Among the 296 *Enterococcus* spp. obtained from fecal samples of black capuchin monkeys, 201 (67.90%) were nonsusceptible to at least one antibiotic evaluated (**Figure 2**). Nonsusceptible to rifampicin (46%), tetracycline (26%), erythromycin (22%), and quinolones (ciprofloxacin/norfloxacin) (19%) was commonly observed, whereas nonsusceptible to nitrofurantoin, chloramphenicol, and ampicillin was observed only in 13%, 0.3%, and 0.3% of the strains, respectively. Further, all isolates were susceptible to vancomycin, streptomycin, gentamycin, and linezolid (**Table 2**). Chi-squared testing showed significant differences (*p* ≤ 0.05) in tetracycline-nonsusceptible strains isolated from wild black capuchin monkeys from SSC when compared to the other groups.

**Table 2:** Antibiotic resistance patterns among enterococci recovered from fecal samples and rectal of wild and captive black capuchin monkeys (*Sapajus nigritus*).

		Number (Percentage) of Nonsusceptible^1^ Strains to:							Profiles			
	Species (n)	AMP^∗^	QUI^∗,1^	CHL^∗^	ERY^∗^	NIT^∗^	RIF^∗^	TET^∗^		SR^∗∗^	DR^∗∗^	MDR^∗∗^
SSC	*E. faecalis* (44)	0	10 (23)	0	9 (20)	0	26 (59)	3 (7)		12 (27)	8 (18)	6 (14)
	*E. hirae* (49)	0	0	0	4 (8)	4 (8)	22 (45)	27 (55)		12 (24)	12 (24)	7 (14)
	*E. faecium* (26)	0	18 (69)	0	16 (61)	7 (27)	11 (42)	23 (88)		5 (19)	7 (27)	14 (54)
	*E. durans* (7)	1 (14)	0	0	1 (14)	4 (57)	7 (100)	1 (14)		3 (43)	2 (29)	2 (29)
	*E. casseliflavus* (9)	0	1 (11)	0	2 (22)	0	4 (44)	0		3 (33)	2 (22)	0
	*E. raffinosus* (1)	0	0	0	0	0	0	0		0	0	0
	*E. avium* (1)	0	0	0	0	0	0	0		0	0	0
	Subtotal (137)	1 (0.7)	29 (21)	0	32 (23)	15 (11)	70 (51)	54 (39)		35 (26)	31 (23)	29 (21)
SCS	*E. faecalis* (77)	0	18 (23)	0	14 (18)	0	41 (53)	7 (9)		36 (47)	16 (21)	4 (5)
	*E. hirae* (2)	0	0	0	0	0	0	0		0	0	0
	*E. faecium* (2)	0	2 (100)	0	1 (50)	0	1 (50)	2 (100)		0	0	2 (100)
	*E. casseliflavus* (4)	0	0	0	0	0	2 (50)	0		2 (50)	0	0
	*Enterococcus sp.* (1)	0	0	0	0	0	0	0		0	0	0
	Subtotal (86)	0	20 (23)	0	15 (17)	0	44 (51)	9 (10)		38 (44)	16 (19)	6 (7)
ZOO	*E. faecalis* (5)	0	2 (40)	0	1 (20)	0	5 (100)	4 (80)		0	3 (60)	2 (40)
	*E. hirae* (35)	0	2 (6)	0	7 (20)	15 (43)	8 (23)	6 (17)		7 (20)	6 (17)	4 (11)
	*E. faecium* (19)	0	2 (10)	1 (5)	10 (53)	2 (11)	3 (16)	3 (16)		6 (32)	4 (21)	2 (10)
	*E. durans* (13)	0	0	0	1 (8)	8 (61)	7 (54)	1 (8)		7 (54)	4 (31)	0
	*E. gallinarum* (1)	0	1 (100)	0	0	0	0	0		1 (100)	0	0
	Subtotal (73)	0	7 (10)	1 (1)	19 (26)	25 (34)	23 (31)	14 (19)		21 (29)	17 (25)	8 (11)
	Total (296)	1 (0.3)	56 (19)	1 (0.3)	66 (22)	40 (13)	137 (46)	77 (26)		94 (32)	64 (22)	43 (14)


*^1^Intermediate and resistant strains were considered in a single category and classified as antibiotic-nonsusceptible. ^∗^Antibiotics: AMP, ampicillin; QUI, quinolones (ciprofloxacin and norfloxacin); CHL, chloramphenicol; ERY, erythromycin; NIT, nitrofurantoin; RIF, rifampicin; TET, tetracycline; ^∗∗^Profiles: SR, single resistant; DR, double resistant; MDR, multidrug-resistant.*

*SSC, São Sebastião do Caí; SCS, Santa Cruz do Sul; ZOO, Sapucaia do Sul.*

In relation to species isolated from black capuchin monkeys, *E. durans* (90%) and *E. faecium* (85%), showed elevated frequency of antibiotic non-susceptibility, followed by *E. faecalis* (69%), *E. hirae* (56%), and *E. casseliflavus* (54%). *Enterococcus gallinarum* strain was only nonsusceptible to quinolones. Unlike the other species, *E. raffinosus*, *E. avium*, and *Enterococcus spp.* were susceptible to all antimicrobials tested. Regarding the source of samples, the occurrence of antibiotic non-susceptible strains was observed more frequently in isolates from SSC (**Figure 2**).

Single, double, and MDR profiles were observed in 32% (*n* = 94), 22% (*n* = 64), and 14.52% (*n* = 43) of strains, respectively. The percentages of double and MDR strains isolated from wild monkeys from SCS (10%; *n* = 9 and 7%; *n* = 6) and the captive (16%; *n* = 12 and 11%; *n* = 8) were lower compared to wild monkeys from SSC (39%; *n* = 54 and 21%; *n* = 29). Among the 29 MDR strains from SSC, *E. faecium* was the species with higher prevalence (54%; *n* = 14) (**Supplementary Data 2**).

### Frequency of Antibiotic Resistance Genes

Among the 66 erythromycin-nonsusceptible strains (11 were resistance and 56 were intermediate resistance), 24 (36%) contained the *msrC*, and none the *erm*(B) gene. Of the 77 tetracycline-nonsusceptible strains, 43 (56%) harbored only the *tet*(M), and 24 (31%) have both *tet*(M) and *tet*(L) genes. The *tet*(S) gene was not found in this study (**Table 3**).

**Table 3:** Resistance-related genes among antibiotic-nonsusceptible enterococci isolated from fecal samples of wild and captive black capuchin monkeys (*Sapajus nigritus*).

		Antibiotic-Nonsusceptible Strains Tested to:									
		Erythromycin					Tetracycline				
	Strains	*R*^1^	*I*^1^	*n*^2^ (%) of *msr*C	*n*^2^ (%) of *erm*(B)		*R*^1^	*I*^1^	*n*^2^ (%) of *tet*(L)	*n*^2^ (%) of tet(M)	*n*^2^ (%) of *tet*(S)
SSC	*E. faecalis*	1	8	0	0		3	0	1 (33)	3 (100)	0
	*E. hirae*	2	2	0	0		20	7	14 (52)	26 (96)	0
	*E. faecium*	0	16	14 (87.5)	0		21	2	0	16 (70)	0
	*E. durans*	1	0	0	0		1	0	0	0	0
	*E. casseliflavus*	0	2	0	0		0	0	ND	ND	0
	Subtotal	4	28	14 (44)	0		45	9	15 (28)	45 (83)	0
SCS	*E. faecalis*	0	14	1 (7)	0		7	0	1 (14)	7 (100)	0
	*E. faecium*	0	1	1 (100)	0		2	0	0	2 (100)	0
	Subtotal	0	15	2 (13)	0		9	0	1 (11)	9 (100)	0
ZOO	*E. faecalis*	0	1	0	0		4	0	0	4 (100)	0
	*E. hirae*	4	3	7 (100)	0		6	0	5 (83)	6 (100)	0
	*E. faecium*	4	6	10 (100)	0		3	0	3 (100)	3 (100)	0
	*E. durans*	1	0	1 (100)	0		1	0	0	0	0
	Subtotal	9	10	18 (95)	0		14	0	8 (57)	13 (93)	0
	Total	11	53	34 (51.5)	0		68	9	24 (31)	67 (87)	0


*^1^Number of resistant (R) or I, Intermediate resistant (I) strains.*

*^*2*^Number of positive strains; ND, not determined; SSC, São Sebastião do Caí; SCS, Santa Cruz do Sul; ZOO, Sapucaia do Sul.*

In relation to species, the results showed that 92.5% *E. faecium*, 64% *E. hirae*, and 4% *E. faecalis* strains harbored *msrC* gene. The *tet*(M) was present in all *E. faecalis*, *E. faecium*, and *E. hirae* tetracycline-nonsusceptible strains, and *tet*(L) was detected in 14% *E. faecalis*, 57.5% *E. hirae*, and in 11% *E. faecium* tetracycline-nonsusceptible strains.

We investigated the association between resistance-related genes and the sample sources where enterococci species isolated from captive monkeys presented a higher frequency of *msr*C (95%) and *tet*(L) (57%) genes when compared to wild monkeys (**Table 3**). In addition, seven (21%) erythromycin and tetracycline-nonsusceptible strains from the ZOO harbored both *msr*C, *tet*(M), and *tet*(L) genes.

## Discussion

In this study using fecal samples collected of wild and captive black capuchin monkeys (*S. nigritus*) from South Brazil, we were able to detected different *Enterococcus* species. To date, only a few studies have investigated the distribution of enterococci species in the fecal samples/rectal swabs of wild and captive black capuchin monkeys. The genus *Enterococcus* was first reported in fecal samples from captive capuchin monkeys (*Cebus apella*) and common marmoset (*Callithrix penicillata*) in the Primate Center of the University of Brasília, Brazil (Xavier et al., 2010). Thereafter, Glover (2014) identified the genus *Enterococcus* in the fecal samples from the baboons (*Papio*) and vervet monkeys (*Chlorocebus pygerythrus*) in two rehabilitation centers in South Africa.

The enterococci species identified here from both wild and captive black capuchin monkeys have been reported to be predominant in fecal samples of different animals. Studies evaluating enterococci species in fecal samples of domestic and wild animals revealed presence of similar species (Layton et al., 2010; Cassenego et al., 2011; Franz et al., 2011; Silva et al., 2012; Nowakiewicz et al., 2014; Santestevan et al., 2015; Prichula et al., 2016; Medeiros et al., 2017). Among the species identified in the present study, *E. faecalis* was predominant. This species was also the most prevalent species in fecal samples of captive capuchin monkeys, common marmoset, domesticated mammals, birds, and wildlife feces, described in previous studies (Lanthier et al., 2010; Xavier et al., 2010). Nevertheless, it is important to highlight that some species could be underestimated in the present study due to the limitation of the method on used for enterococci isolation based on culturable methods. Although this method is widely used to isolate enterococci from different samples; we know that methods evaluating bacterial species in biological samples based on cultivation could limit the ability to recover some species occurring in small proportion.

Differences in the frequency of enterococci species in fecal samples among the three groups of monkeys were observed. Confinement, diet, and human contact are factors that may be responsible for this difference (Lebreton et al., 2014). In fecal samples of wild monkeys from SCS, the *E. faecalis* was the dominant species. In contrast, the species distribution of enterococci in samples of wild monkeys from SSC was more heterogeneous. These differences in the frequency of enterococci could be explained by the environmental conditions. In spite of the fact that both monkeys live in a free-living condition, monkeys from SCS are in a forest fragment surrounded by an urban area. Urban forest fragments are considered the most fragile area, which suffers directly the negative impacts of the anthropic action (Pereira et al., 2018). The urbanization also affects the insect species composition, as recently demonstrated by Melliger et al. (2018), whereas changes in the composition of ants and spiders were associated with increasing degree of urbanization. The anthropic action on the forest fragment in SCS may have reduced the contact of monkeys with diverse routes transmitting variable enterococci, including insect that comprised approximately 33% of the diet of these animals. Besides, the monkeys from SCS are feeding by human and have access to the garbage left by visitors on the park.

Contrasting with wild monkeys from SCS, the fecal samples from wild monkeys of SSC showed more dissimilar *Enterococcus* species, including *E. faecalis*, *E. hirae*, *E. faecium*, *E. durans*, *E. casseliflavus*, *E. raffinosus*, *E. avium*, and *E. gallinarum*. These monkeys live in a less urbanized forest fragment with a general diet, composed by insects, fruits, stems, flowers, and leaves, and consequently exposed to several *Enterococcus* species. In captive monkeys from ZOO, which are feeding with nonhuman dry food – composed by proteins, crude fiber and fat – supplemented with fruits and vegetables, the *E.* *hirae*, *E. faecium*, and *E. durans* were the most prevalent species. The presence of these *Enterococcus* species might be associated with the food source since enterococci were detected in the feed and feed ingredients samples as described by da Costa et al. (2007) and Ge et al. (2013).

Antibiotic-nonsusceptible enterococci species were found in captive and black capuchin monkeys. Similar studies, detected resistant bacteria in captive and wild animal from different environments (Xavier et al., 2010; Santos et al., 2013; Glover, 2014; Smith et al., 2014; Santestevan et al., 2015; Bondarczuk et al., 2016; Prichula et al., 2016; Furness et al., 2017; Bengtsson-Palme et al., 2018). In addition, samples from wild black capuchin monkeys from SSC presented a high number of antibiotic-nonsusceptible strains. The antibiotic-nonsusceptible strains isolated from wild monkeys are a matter of concern since these animals did not have a history of therapeutic antibiotic exposure. The analysis of resistant enterococci in these animals emphasizes the role of human activities on the environment. However, we cannot forget to mention that wild black capuchin monkeys from SSC live in a forest fragment near a public hospital and water treatment plant, and this proximity with these environments should represent a source of antibiotic-nonsusceptible strains in these animals. The presence of bacteria antibiotic-nonsusceptible and antibiotic resistance genes in hospital effluents has been observed and related to dissemination of resistance in the environment (Brown et al., 2006; Rodríguez-Mozaz et al., 2014; Xu et al., 2014). For example, tetracycline and erythromycin prescribed in human and animal medicine are excreted as active metabolites and remain stable in the environment (Rahardjo et al., 2011; Rudra et al., 2018; Schafhauser et al., 2018) to be considered modern pollutants in soils and aquatic environment (Gothwal and Shashidhar, 2014; Dizavandi et al., 2016). Another aspect to be considered is the antibiotic resistome (Wright, 2007; Stewart et al., 2014). Previous reports have noted the occurrence of resistant bacteria in soil independent of human activity (Allen et al., 2010). As such, we cannot exclude the possibility that the resistance found in monkeys is derived from the gut microbial communities. Tsukayama et al. (2018) showed that antibiotic resistance is an ancient feature of gut microbial communities of primate and that sharing habitats with humans may have an important impact on the structure and function of this microbiota.

In our study, 14% of the isolated strains were resistant at least to three or more drugs. The MDR enterococci species have been isolated from wild and captive animals (Nowakiewicz et al., 2014; Prichula et al., 2016). It is important to note that an elevate number of MDR *E. faecium* isolated from wild monkey that lives near to the hospital was detected. In the last years, the emergence of MDR bacteria has become a hospital-acquired infection problem and, a high number of MDR enterococcal infections are caused by *E. faecium* (Kristich et al., 2014).

The resistance-related genes commonly observed in this environment, *msr*C, *tet*(M), and *tet*(L) was detected in our samples. Those resistance genes were found in higher frequency in samples from captive monkeys when compared to wild monkeys. Perhaps, the captive condition of animals might be contributing to the acquisition/dispersion and persistence of these genes in this environmental. Up until now, only two studies have evaluated resistant genes in enterococci-resistant isolated from monkeys (Xavier et al., 2010; Woods et al., 2017). Our data demonstrated the *tet*(M) gene is widely distributed among our isolates followed by *tet*(L). Furthermore, when studying enterococci from wild marine animals, Prichula et al. (2016) identified a high prevalence (73.07%) of the *tet*(M) gene and a low prevalence (23.07%) of *tet*(L), which corroborates with the findings in the present study. Notably, Poeta et al. (2005) determined that the *tet*(M) gene is the more prevalent in enterococci from wild animals in Portugal, other than monkeys. Moreover, 50% of samples from Santestevan et al. (2015), isolated from wild sea lions presented *tet*(M) gene. Despite *erm*(B) gene is frequently observed in macrolide-resistant strains isolated from animals (Poeta et al., 2005; Cassenego et al., 2011), this gene was not detected in our samples. In addition, the *msr*C gene was detected at low frequency in wild monkeys. Additionally, Prichula et al. (2016) tested the *erm*(B) and *msr*C in enterococci strains isolated from wild marine animals and reported only the presence of the *msr*C. It is possible that other genes could be associated with erythromycin-nonsusceptible strains isolated from monkeys, like *erm*(A)*, erm*(C), *erm*(D), *erm*(E), *erm*(F), *erm*(G), *erm*(Q), and the macrolide efflux pump (*msrA*).

In conclusion, the enterococci isolated in this study from monkeys living in three distinct areas, showed differences in the species, in the frequency of antibiotic-nonsusceptible and antibiotic resistance genes. These differences could be related to food web interactions, environmental pollutants, and/or antibiotic resistome. High frequency of MDR strains was observed in fecal samples of wild monkeys, which live in a forest fragment near a public hospital. The data presented in this study suggest that anthropogenic action might be affecting primate-gut enterococci community.

Finally, further research is necessary to better understand the evolution of resistance mechanisms presented by enterococci. Therefore, this study contributes in part to the comprehension of black capuchin monkey’s microbiota, and to the elucidation of resistant bacterial strains and spread in wild and captive environments.

## Author Contributions

TG, JF, PW, and AF designed the study. RP and RS performed the MIC. TG, DZ, PW, and AA carried out the sampling work. TG, LC, JF, and AF analyzed the data and drafted the manuscript. All authors have read and approved the final manuscript.

## Conflict of Interest Statement

The authors declare that the research was conducted in the absence of any commercial or financial relationships that could be construed as a potential conflict of interest.
