# Supplementary material for: Antimicrobial Resistance Profiles in Enterococcus spp. Isolates From Fecal Samples of Wild and Captive Black Capuchin Monkeys (Sapajus nigritus) in South Brazil
Source: Front Microbiol. 2018 Oct 9;9:2366. doi: 10.3389/fmicb.2018.02366 (PMC6189294; doi:10.3389/fmicb.2018.02366)
Supplement: Supplementary file 1 [file Table_1.docx]

Supplementary Material

**ANTIMICROBIAL RESISTANCE PROFILES IN *Enterococcus* spp. ISOLATES FROM FECAL SAMPLES OF WILD AND CAPTIVE BLACK CAPUCHIN MONKEYS (S*apajus nigritus*) IN SOUTH BRAZIL**

**Tiela Trapp Grassotti, Dejoara de Angelis Zvoboda, Letícia da Fontoura Xavier Costa, Alberto Jorge Gomes de Araújo, Paulo Guilherme Carniel Wagner, Jeverson Frazzon, Ana Paula Guedes Frazzon^*^**

*** Correspondence:** Corresponding Author: ana.frazzon@ufrgs.br

**Supplementary Data**

1. Details of the black capuchin monkeys **(***Sapajus nigritus*) analyzed in this study

|  | **Location** | **ID^1^** | **Sample source** | **Age** | **Sex** | **Weight (g)** |
| --- | --- | --- | --- | --- | --- | --- |
| Wild | São Sebastião do Caí (SSC) | SN-1 | Fecal sample (individual) | ND | ND | ND |
|  |  | SN-3 | Fecal sample (individual) | ND | ND | ND |
|  |  | SN-4 | Fecal sample (individual) | ND | ND | ND |
|  |  | SN-9 | Rectal swab | Juvenile | Female | 1.52 |
|  |  | SN-10 | Rectal swab | Adult | Female | 3.03 |
|  |  | SN-11 | Rectal swab | Adult | Female | 2.40 |
|  |  | SN-12 | Rectal swab | Adult | Female | 2.07 |
|  |  | SN-13 | Rectal swab | Juvenile | Female | 1.90 |
|  |  | SN-14 | Rectal swab | Juvenile | Male | 1.56 |
|  |  | SN-15 | Rectal swab | Juvenile | Male | 2.20 |
|  |  | SN-17 | Fecal samples (pool) | ND | ND | ND |
|  | Santa Cruz do Sul  (SCS) | SN-21 | Fecal samples (pool) | ND | ND | ND |
|  |  | SN-22 | Fecal samples (pool) | ND | ND | ND |
|  |  | SN-24 | Rectal swab | Adult | Male | 3.17 |
|  |  | SN-25 | Rectal swab | Juvenile | Male | 1.90 |
|  |  | SN-26 | Rectal swab | Adult | Female | 2.27 |
|  |  | SN-27 | Rectal swab | Adult | Male | 2.00 |
|  |  | SN-28 | Rectal swab | Adult | Male | 2.20 |
|  |  | SN-29 | Fecal samples (pool) | ND | ND | ND |
| Captive | Sapucaia do Sul (ZOO) | SN-16 | Fecal sample (individual) | ND | ND | ND |
|  |  | SN-18 | Fecal sample (individual) | ND | ND | ND |
|  |  | SN-19 | Fecal sample (individual) | ND | ND | ND |
|  |  | SN-20 | Fecal sample (individual) | ND | ND | ND |
|  |  | SN-30 | Fecal samples (pool) | ND | ND | ND |

1. ID: Sample identification. ND – Not disponible

**2.** Multidrug resistant profiles of enterococci recovered from fecal samples of wild and captive black capuchin monkeys **(***Sapajus nigritus*)

| **MDR** | **SSC (n)** | **SCS (n)** | **ZOO (n)** |
| --- | --- | --- | --- |
| QUI/ERI/RIF | *E. faecalis* (3) | *E. faecalis* (4) | *E. faecalis* (1); *E. faecium* (1) |
| ERI/RIF/TET | *E. faecalis* (1); *E. hirae* (3) |  |  |
| NIT/RIF/TET | *E. hirae* (4) |  |  |
| QUI /RIF/TET | *E. faecium* (1) | *E. faecium* (1) | *E. faecalis* (1) |
| AMP/NIT/RIF | *E. durans* (1) |  |  |
| QUI /ERI/TET |  | *E. faecium* (1) |  |
| ERI/NIT/TET |  |  | *E. hirae* (1) |
| QUI /NIT/RIF |  |  | *E. hirae* (1) |
| QUI /ERI/NIT |  |  | *E. hirae* (1) |
| QUI /ERI/TET | *E. faecium* (3) |  |  |
| CIP/ERI/NIT/TET | *E. faecium* (1) |  |  |
| ERI/NIT/RIF/TET | *E. durans* (1) |  | *E. hirae* (1) |
| ERI/NIT/ QUI /TET | *E. faecium* (1) |  |  |
| QUI /ERI/RIF/TET | *E. faecalis* (2); *E. faecium* (4) |  | *E. faecium* (1) |
| QUI /ERI/NIT/RIF/TET | *E. faecium* (3) |  |  |
| QUI /ERI/NIT/RIF/TET | *E. faecium* (1) |  |  |
| **Total** | **29** | **6** | **8** |

*Antibiotics: AMP: Ampicillin; QUI: quinolones (ciprofloxacin and norfloxacin); CHL: chloramphenicol; ERY: erythromycin; NIT: nitrofurantoin; RIF: rifampicin; TET: tetracycline.

SSC: São Sebastião do Caí; ZOO: Sapucaia do Sul

**
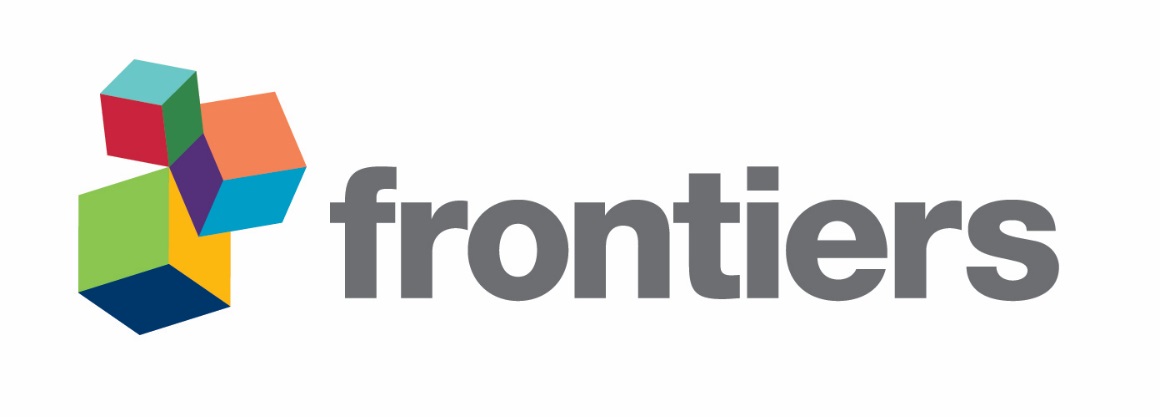
**
